# Noninvasive early detection of anthracycline‐induced cardiotoxicity in patients with hematologic malignancies using the phased tracking method

**DOI:** 10.1002/cam4.813

**Published:** 2016-08-03

**Authors:** Yoshiko Saito, Ikuko Susukida, Yoshiro Uzuka, Hiroshi Kanai

**Affiliations:** ^1^ Department of Food and NutritionMiyagigakuin Women's UniversitySendaiJapan; ^2^ Sendai Blood Disease CenterSendaiJapan; ^3^ Graduate School of Biomedical Engineering and the Graduate School of EngineeringTohoku UniversitySendaiJapan

**Keywords:** Acute leukemia, anthracycline, cardiotoxicity, echocardiography, malignant lymphoma, phased tracking method

## Abstract

Anthracyclines are among the most effective and widely used anticancer drugs; however, their use is limited by serious cardiotoxicity. Early detection is necessary to prevent the high mortality rate associated with heart failure (HF). We evaluated cardiac function in 142 patients using conventional echocardiography and the phased tracking method (PTM), which was measured using the minute vibration and the rapid motion components, neither of which is recognized in standard M‐mode nor in tissue Doppler imaging. For systolic function comparison, we compared left ventricular ejection fraction (LVEF) in conventional echocardiography with the average velocity of ventricular septum myocytes (V_ave_) in the PTM. The V_ave_ of 12 healthy volunteers was 1.5 (m/s)/m or more. At baseline of 99 patients, there was a positive correlation between LVEF and V_ave_ in all patients. There were no significant differences in baseline cardiac function between patients with and without HF. There was a negative correlation between the cumulative anthracycline dose and LVEF or V_ave_ among all patients. We determined that V_ave_ 1.5 (m/s)/m was equivalent to LVEF 60%, 1.25 (m/s)/m to 55%, and 1.0 (m/s)/m to 50%. During the follow‐up period, there was a pathological decrease in LVEF (<55%) and V_ave_ (<1.25 m/s/m) in patients with HF; decreases in V_ave_ were detected significantly earlier than those in LVEF (*P* < 0.001). When V_ave_ declined to 1.5 (m/s)/m or less, careful continuous observation and cardiac examination was required. When V_ave_ further declined to 1.0 (m/s)/m or lower, chemotherapy was postponed or discontinued; thus, serious drug‐induced cardiomyopathy was avoided in patients who did not relapse. The PTM was superior to echocardiography for early, noninvasive detection and intermediate‐term monitoring of left ventricle systolic function associated with anthracycline chemotherapy, among patients with hematologic malignancies. The PTM was an effective laboratory procedure to avoid the progression to serious cardiomyopathy.

## Introduction

Anthracycline treatment significantly improves the survival of patients with cancer, but can result in serious cardiotoxicity; therefore, effective monitoring of anthracycline‐induced changes in cardiac function could detect changes early enough to implement treatment strategies to significantly reduce or prevent such life‐threatening complications 1. The development of noninvasive, reproducible, easy‐to‐use, sensitive diagnostic techniques would greatly improve the detection of early pathological changes associated with treatment regimens 2, 3. One such technique is the phased tracking method (PTM) 4, a high‐resolution Doppler measurement that is useful for detecting cardiac transmural changes 5, 6, the PTM is measured on minute amplitude on the order of several tens of micrometers up to several hundred Hertz, which has neither been recognized in TDI nor standard M‐mode, B‐mode, 2D‐strain echocardiography. These fine measurement characteristics and the rapid minute amplitude have allowed measurement of the fetal heart and the fetal descending aorta of normal and growth‐restricted fetuses 7, 8.

The aim of this study was to compare changes in left ventricle cardiac parameters detected using the PTM with those obtained using conventional echocardiography in order to evaluate whether the PTM is superior for the detection of early anthracycline‐induced changes in cardiac function in patients with acute lymphocytic leukemia (ALL), acute myeloid leukemia (AML), and malignant lymphoma (ML). We had experienced a case on the basis of the PTM measurement data, by adjusting the chemotherapy schedule as the development of serious cardiomyopathy was prevented 9 and improvement of prognosis is expected.

## Methods

### Study population

Twelve healthy volunteers and 142 patients with hematologic malignancies were eligible for the PTM and conventional echocardiography. Between August 1998 and August 2014, 128 consecutive patients underwent echocardiography and PTM to evaluate cardiac function. An additional 14 AML patients were examined after completion of chemotherapy in 2009. Patients were grouped according to the absence or presence of clinical heart failure (HF), no HF group and HF group, respectively (Table 1). Informed consent was obtained from all patients.

**Table 1:** Characteristics of patients who underwent echocardiography (*n* = 142)

	No. pt.	No HF group (*n* = 118)				HF group (*n* = 24)			
		ALL	ML	AML	No. pt.	ALL	ML	AML	No. pt
Number of patients	142	13	20	85		1	13	10	
Male	72	4	14	43	61	0	7	4	11
Female	70	9	6	42	57	1	6	6	13
Age at diagnosis, years									
Average	58	51.5	50.6	54.7		21	56.8	47.8	
Median	62	51	48	58		/	55	54	
Range	16–89	18–82	18–80	14–88		/	41–74	18–69	
Current age*, years									
Average	53	53	56.6	62.7		27	64	54.2	
Median	54	50	57	66		/	60	56	
Range	14–86	18–82	20–84	15–93		/	46–87	21–79	

HF, heart failure; AML, acute myeloid leukemia; ALL, acute lymphocytic leukemia; ML, malignant lymphoma, *, Final inspection age.

### Chemotherapeutic protocols

Patients with ALL or ML underwent the modified CHOP protocol 10, 11, whereas those with AML underwent therapy with daunorubicin and cytosine‐arabinoside 12, 13, 14. Daunorubicin doses were converted to doxorubicin equivalents using a conversion factor of 0.56 that was the proposed equivalent tumor effect doses obtained with a standard protocol.

### Echocardiography

Patients underwent comprehensive two‐dimensional and Doppler echocardiographic examinations, which were performed by a single doctor using a EUB 655 ultrasound scanner (Hitachi, Ibaraki, Japan) and a SSD‐500SV ultrasound scanner (ALOKA, Tokyo, Japan) in accordance with recognized standards. Left ventricular ejection fraction (LVEF) was calculated using modified Teichholz.

### Phased tracking method

**Figure 1:**
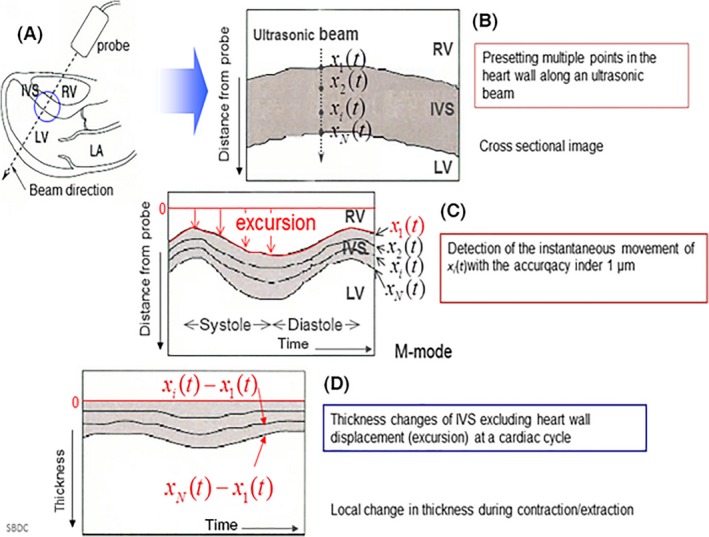
Process of the evaluation of the local change in the thickness during one cardiac cycle.

**Figure 2:**
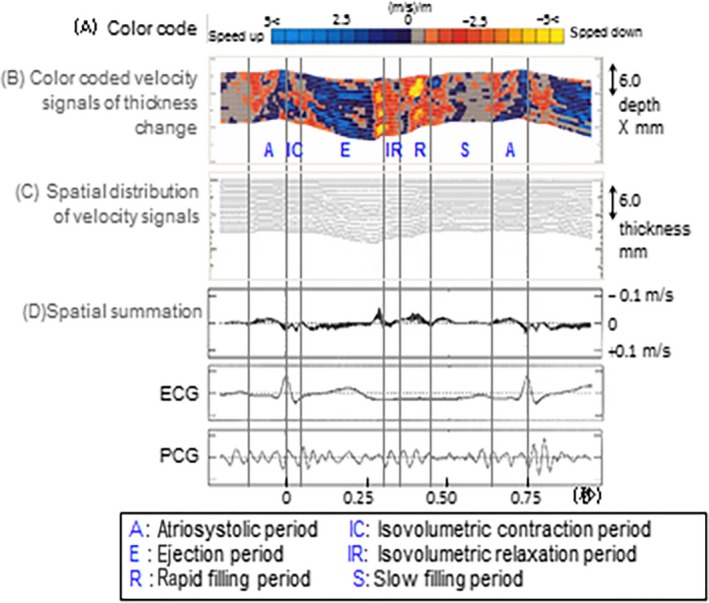
Formula to calculate the average velocity (Vave) of cardiomyocytes in the interventricular septum during opening and closing of the aortic valve.

The ultrasound scanner was switched to the phased tracking mode. By referring to the M‐mode image, which was constructed from the analog/digital (A/D) converted data, we manually preset two points, in the heart wall, between which, the ultrasonic beam was directed, as illustrated in Figure 1A. The principles of the PTM are illustrated in Figure 1B–D. Using the M‐mode PTM as the parasternal long‐axis view, we measured the M‐mode image, electrocardiography, phonocardiogram, and small‐amplitude velocity signals of less than a few micrometers of the interventricular septum (IVS) by tracking the results of the multiple points that normalized the speed of the change in thickness. Because the results obtained by the proposed method depend on the angle between the direction of the velocity vector and the ultrasonic beam, the direction of the ultrasonic beam passing through heart wall was selected, so that the beam was almost perpendicular to each wall during the A/D conversion of several cardiac cycles. During the acquisition period, respiration was suspended. Figure 2 shows the superimposed estimates of the velocity signals {*v* ‐ (*x*_*i*_; *t*)} of each heart beat on the tracked points {*x*_*i*_ ‐ (*t*)} on IVS during six heartbeats. The vertical axis of these figures was inverted so that the negative value of the velocity, which is shown above the baseline, corresponds to the situation in which the object moves in the direction of the ultrasonic transducer on the chest wall. The resultant velocity signals are sufficiently reproducible for six heartbeat periods. The results were immediately visualized and output as shown in Figure 2.

### Statistical analyses

The results are presented by the mean and median values. Paired and nonpaired two‐tailed Student's *t*‐tests were used to compare parameters between the groups using the JMP11 statistical software (SAS Institute Inc., Cary, NC).

## Results

**Figure 3:**
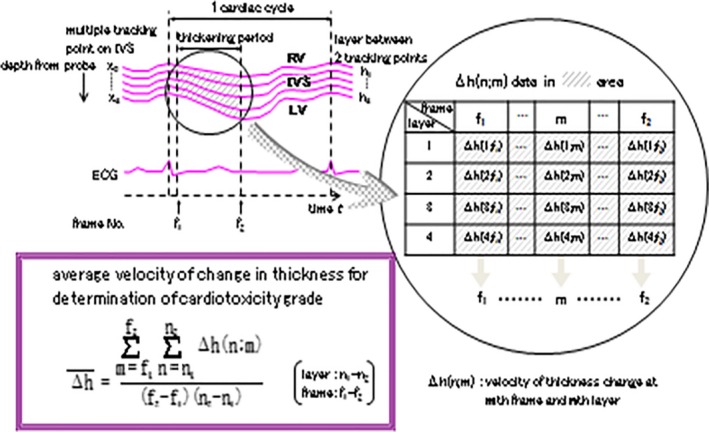
Average velocity of change in thickness for determination.

The contractile force and contraction synchrony of myocardial cells was measured using the PTM. We analyzed the period between aortic valve opening and closing (as shown in Fig. 3) and calculated the average velocity (V_ave_) using the following equation:

where V_max_ is the highest velocity of the layers.

$$\bar{\Delta h}=\frac{\sum_{m=f_{1}}^{f_{2}}\sum_{n=n_{1}}^{n_{2}}\Delta h\left(n;m\right)}{\left(f_{2}-f_{1}\right)\left(n_{2}-n_{1}\right)}$$

Of the 142 patients who underwent echocardiographic evaluations, 128 patients were sequentially examined during and after the completion of chemotherapy; however, patients sometimes missed examinations for deconditioning. Fourteen patients were evaluated only once, ten or more years long after completion of chemotherapy. Characteristics of the 142 patients who underwent echocardiographic evaluations are shown in Table 1. Patients were divided into two groups; no clinical cardiac symptoms (no HF group and cardiac clinical symptoms HF group). HF occurred in 11 male patients and 13 female patients, respectively (odds ratio 0.79 95% CI: 0.33–1.91). There were no significant differences between patient characteristics in the ALL, AML, and ML groups, with the exception of the mean age of patients with ALL, which was 21 years at the time of diagnosis, approximately 30 years younger than the average age of patients in the other groups.

**Figure 4:**
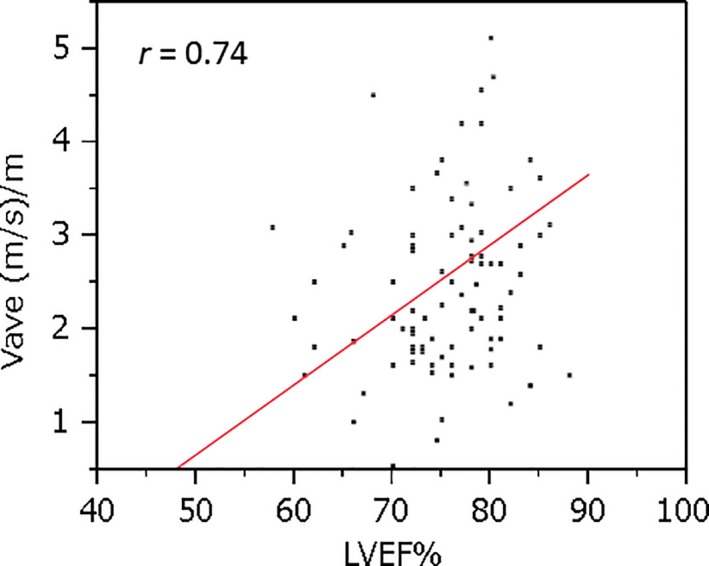
Correlation with left ventricular ejection fraction and average velocity at baseline.

Prior to treatment, baseline LVEF was positively correlated with V_ave_ (*r* = 0.746**8;** Fig. 4). One hundred and eighteen patients had no clinical HF, with a maximum anthracycline dose of 2205 mg/m^2^. Among the 24 patients in HF group, the minimum cumulative anthracycline dose associated with development of HF was 241 mg/m^2^. There were no significant differences in baseline cardiac function between no HF group and HF group (Table 2).

**Table 2:** Baseline cardiac functions between patients with no clinical cardiac symptoms (no heart failure [HF] Group) and those with congestive heart failure clinical symptoms (HF Group)

Echocardiography	No HF groupMean average ± SD	HF groupMean average ± SD	*P*
CTR %	47 ± 0.06	46.3 ± 0.04	0.5
QTC, sec	0.42 ± 0.039	0.4225 ± 0.026	0.6
IVC, mm	11.12 ± 2.86	10.7 ± 1.85	0.62
AO, mm	19.64 ± 3.14	19.7 ± 2.21	0.64
LA mm	30 ± 5.09	29.7 ± 3.52	0.52
LVEF	0.753 ± 0.063	0.76 ± 6.6	0.84
FS	0.374 ± 0.055	0.378 ± 5.4	0.06
PEP/ET	0.253 ± 0.119	0.31 ± 0.082	0.96
A/E	0.884 ± 0.272	0.84 ± 0.386	0.7
The PTM			
Vave (m/s)/m	2.427 ± 0.971	2.39 ± 0.972	0.94
Ths/thd	2.14 ± 071	2.06 ± 0.676	0.82
Vmax (m/s)/m	21.89 ± 17.64	24.7 ± 17.64	0.09

AO, aortic valve diameter; CTR, cardiothoracic ratio; E/A, left ventricular flow; FS, fractional shortening; IVC, inferior vena cava diameter; LA, left atrial diameter; LVEF, left ventricular ejection fraction; PEP/ET, pre‐ejection time/ejection time; PTM, phased tracking method; QTc, correction QT time; SD, standard deviation; Ths/Thd, ratio of thickness in the systolic and in the diastolic phase; V_ave_, average velocity of cardiomyocytes; V_max_, maximum velocity of cardiomyocytes.

Table 3 shows the correlation between the cumulative anthracycline dose and LVEF or V_ave_. Both LVEF and V_ave_ decreased with increasing anthracycline cumulative dose in all patients. However, in HF group, V_ave_ decreased at a significantly smaller cumulative dose point than did LVEF (*P* = 0.003). V_ave_ also significantly decreased 2–3 years after the completion of chemotherapy (*P* = 0.01); however, among patients who survived more than 5 years after the completion of chemotherapy, those in no HF group had recovered cardiac function, whereas cardiac function remained depressed in HF group. In no HF group, LVEF was not shown the reduction but Vave showed decreased (0.001), also, in HF group, Vave was shown a significant declined than LVEF (0.01) after completion of chemotherapy.

**Table 3:** Correlation between the cumulative anthracycline dose and the cardiac functions of left ventricular ejection fraction and average velocity of cardiomyocytes

	No heart failure (HF) group			HF group		
Cumulative DOXDose (mg/m^2^)	LVEF (%)Mean ± SD	Vave ((m/s)/m)Mean ± SD	*n*	LVEF (%)Mean ± SD	Vave((m/s)/m)Mean ± SD	*n*
0	75.3 ± 6.3	2.43 ± 0.97	87	76 ± 6	2.43 ± 0.97	18
100	74 ± 5.9	2.075 ± 0.904	42	73.1 ± 5.1	1.851 ± 0.749	14
200	74.7 ± 6.2	2.14 ± 0.853	46	71.3 ± 6.9	2.080 ± 0.815	16
300	73.5 ± 5.3	2.008 ± 0.637	46	68.8 ± 6.8	1.879 ± 0.676	17
400	73 ± 5.7	2.13 ± 0.665	44	68.8 ± 9.9	1.89 ± 0.924	18
500	68.8 ± 6.4	2.157 ± 0.775	46	66.5 ± 8.2	1.75 ± 0.459	15
600	71 ± 1.1	2.2 ± 0.086	41	61.8 ± 13.6	1.32 ± 0.64	17
Completion of CT after 2–3y	71 ± 10.99	1.82 ± 0.805	45	54 ± 13.9	1.096 ± 0.805	13
Completion of CT after 5or more years	71 ± 9.2	1.99 ± 0.82	50	50.9 ± 13.9	0.95 ± 1.016	10

CT, chemotherapy; DOX, doxorubicin; SD, standard deviation; LVEF, left ventricular ejection fraction; V_ave_, average velocity of cardiomyocytes.

**Figure 5:**
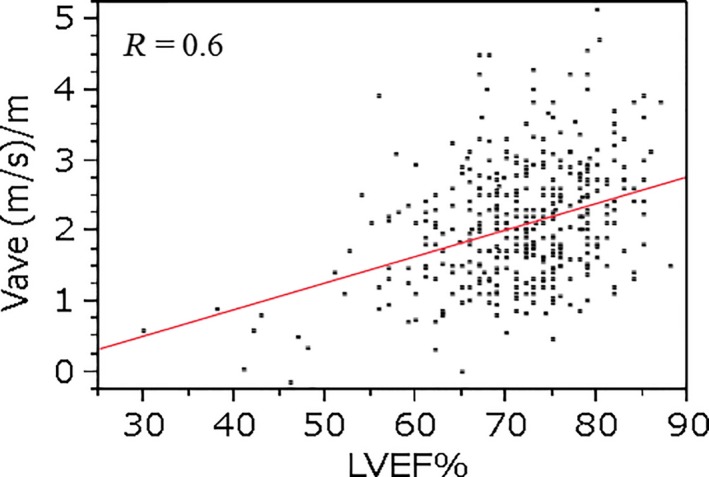
Correlation between the percentage left ventricular ejection fraction (%LVEF) and average velocity of ventricular septum myocytes (V_ave_) during the course of chemotherapy.

In accordance with the historical proposal of cardiotoxicity definition 14, 15, V_ave_ 1.5 (m/s)/m was comparable to LVEF 60%, V_ave_ 1.25 (m/s)/m to LVEF 55%, and V_ave_ 1.0 (m/s)/m to LVEF 50%, at 1 and 12 months before the onset of HF 2, 16 (Fig. 4). As shown in Table 4, V_ave_ declined significantly earlier than did LVEF. Figure 5 shows the correlation between %LVEF and V_ave_ during the courses of chemotherapy. The value of the linear slope in Fig. 5 (r = 0.6) was lower than that in Fig. 4 (r = 0.74). The effect of myocardial disturbance on chemotherapy was more sensitive in terms of the V_ave_ than the LVEF.

**Table 4:** Percentile incidence of left ventricular ejection fraction and average velocity of cardiomyocytes from 1 to 12 months before the onset of congestive heart failure (HF)

Period of up to the onset of congestive HF	1 month before	2 months before	3 months before	6 months before	12 months before	*P*‐value
LVEF < 60%	46.2	60	40	54.5	9	0.048
V_ave _< 1.5 (m/s)/m	73	62.5	80	60	50	
LVEF < 55%	23	40	20	20	9	0.01
V_ave _< 1.25 (m/s)/m	64	62.5	70	60	50	
LVEF < 50%	23	20	20	19	9	0.01
V_ave _< 1.0 (m/s)/m	45.5	62.5	40	40	20	

LVEF, left ventricular ejection fraction; V_ave_, average velocity of cardiomyocytes.

In Table 5, among 12 healthy volunteers, 99 patients before chemotherapy with no heart disease at baseline, and 26 patients with hematologic disease with neither malignancy nor heart disease, the average value of V_ave_ was 2.503 (m/s)/m, with maximum and minimum values of 5.117 (m/s)/m and 0.99 (m/s)/m, respectively).

**Table 5:** Average and maximum velocity of cardiomyocytes and the ejection fraction at baseline, among healthy volunteers and patients

		No of examined cases	Average	Mean	Range	SD
Healthy volunteers	LVEF	12	0.71	0.72	0.6–0.81	0.076
	Vave		2.49	2.28	1.75–12.33	0.96
	Vmax		25.27	21.45	12.33–40.18	9.5
Baseline	LVEF	99	0.76	0.76	0.76–0.88	0.063
	Vave		2.46	2.25	0.54–5.1	0.915
	Vmax		22.22	19.4	5–43.1	12.813
Nonmalignant hematologic diseases	LVEF	26	0.78	0.76	0.86–0.53	0.08
	Vave		2.429	2.2	5.84–1.2	1.154
	Vmax		18.888	20.01	9–35	14.195
Total cases	LVEF	137	0.75	0.76	0.52–0.88	0.07
	Vave		2.482	2.252	0.99–5.835	0.953
	Vmax		22.19	19.7	5.8–76	12.778

LVEF, left ventricular ejection fraction; V_ave_, average velocity of cardiomyocytes; V_max_, maximum velocity of cardiomyocytes.

**Figure 6:**
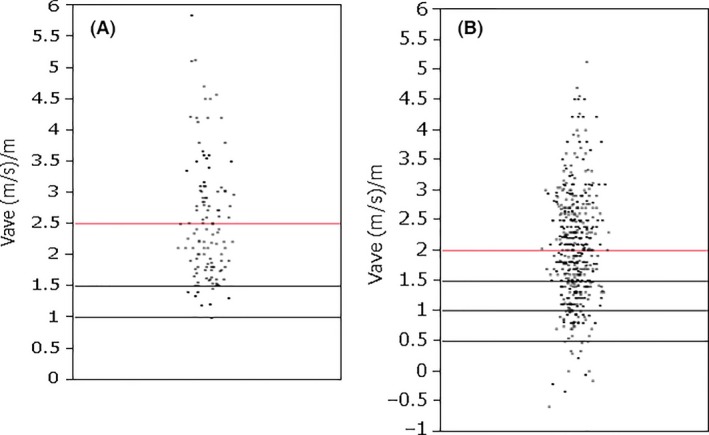
The values of Vave. (A) The average value of Vave of 12 healthy volunteers and 99 patients before chemotherapy was 2.503 (m/s)/m, with maximum and minimum values of 5.117 (m/s)/m and 0.99 (m/s)/m, respectively. (B) The average values of Vave in 520 examinations during the course of the study was 2.012 (m/s)/m, 1.0 (m/s)/m or less in 20 patients, 0.5(m/s)/m or less was 14 patients.

10% had V_ave _≤ 1.5 (m/s)/m and 2.5% had V_ave _≤ 1.0 (m/s)/m (Fig. 6A). Figure 6B shows the values of V_ave_ in 520 examinations during the course of the study. Among this group, 20 of 49 patients (40.8%) with V_ave _≤ 1.0 (m/s)/m developed HF, and 14 of these patients with V_ave _≤ 0.5 (m/s)/m soon developed severe HF.

Table 6 shows data regarding the sensitivity and specificity of V_ave_. Powerful and effective action of the heart is sustained by synchronization of cardiac myocytes. In Figure 2B, the mottled pattern of a visualized color‐coded velocity signal schematic shows disturbance of the continuous smooth movement at the left ventricular septum, during opening and closing of the aortic valve. Cut‐off value setting of faster velocity is needed further study.

**Table 6:** The determination of sensitivity and specificity for the detection of congestive heart failure by the Vave with the phased tracking method

Vave (m/s)/m	Sensitivity	Specificity
1.25	0.98	0.96
1	0.87	0.96
0.5	0.64	1

When V_ave_ values fell to ≤1.5 (m/s)/m, the rest period was extended to allow recovery of at least V_ave_ 1.5 (m/s)/m, then the following treatment was initiated. For cases in which V_ave_ decreased <1.0 (m/s)/m, we canceled additional chemotherapy treatment. Even if myocardial damage is expressed, it is possible to long‐term survival if not experienced recurrence (*P* = 0.0001) in Table 7. Among patients who experienced recurrence, cardiac function decreased and patients developed fatal cardiomyopathy; however, patients who did not relapse did not suffer fatal cardiomyopathy, and some of them recovered 9.

**Table 7:** Survival of patients after V_ave_ decreased below 1.0 (m/s)/m led to the cessation of chemotherapy versus the survival of relapse‐free or relapsed patients

	CaseNumber	Survival time after cessation of chemotherapy			Survival time after relapse		
		Average	Mean	Range	Average	Mean	Range
Relapse‐free patients	6	131 months+	158 months +	52 months +–163 months +	–	–	–
Relapsed patients	18	24 months	17 months	1 months–88 months	4 months	2 months	7 days–17 months
		*P* < 0.0001					

**Figure 7:**
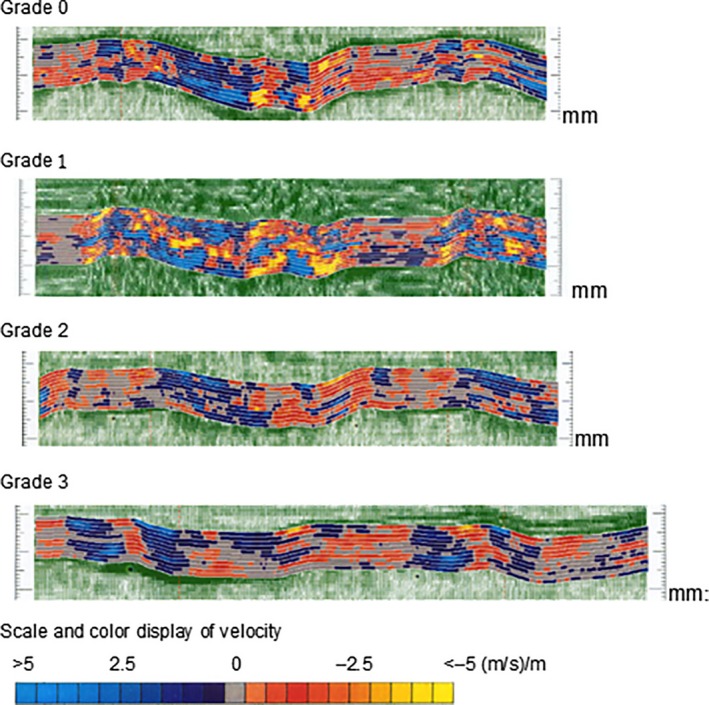
Color‐coded velocity signals to correspond with the Grade; upper the right chamber side, down in the left chamber side, showed the thickness change in the transition rate and the ventricular septal wall of one beat of the heart muscle.

The summary of the aforementioned results and our proposal for clinical treatment of patients in each group are shown in Table 8. Examples of the color‐coded image for each grade of velocity in table 8 are shown in Figure 7.

**Table 8:** The summary of the aforementioned results and our proposal for clinical treatment

	Vave (m/s)/m	Cardiotoxicity	Medical care attitude
Grade 0	≧1.5	No	No
Grade 1	>1.5–≧1.0	Slightly	Careful observation is required
Grade 2	<1.0–≧0.5	Moderate	Treatment postponed or discontinuation is desirable.
Grade 3	<0.5	Severe	Fatal cardiomyopathy is tight.

## Discussion

Anthracyclines were originally introduced in the late 1960s as chemotherapeutic agents. They are extremely effective drugs for the treatment of leukemia and a wide variety of solid tumors. In patients undergoing anthracycline therapy, dose‐dependent myocardial impairment has been described in 23–74% of cases, with a high incidence of HF or fatal complications in 5–19% of cases 2, 3, 17, 18, 19, 20, 21. In contrast, there are no evidence‐based guidelines for monitoring cardiotoxicity in adult patients with cancer 22, 23. Currently, echocardiography and multigated radionuclide angiography are the most commonly used techniques for noninvasive baseline and serial assessment of LVEF 3, 19, 23, 24. Current echocardiography guidelines focus on quantitative measurements of LVEF, rather than visual assessment 2, 17, 18, 19, 22, 25, 26, 27. The fundamentals, strengths, and limitations of these techniques were the topic of a recently published consensus statement 28, 29. The high‐resolution Doppler measurement, showed the systolic heterogeneity of subendocardial myocardial ischemia in normal subjects 1.

The PTM, which was employed in this study, has been applied to clinical studies by many researchers, that is, noninvasive evaluation of the myocardial property of contraction and relaxation 5, 30, 31, elasticity of the arterial wall 32, 33, 34, 35, 36, and fatal cardiac or artery 7, 8, 37. Although in this study, we reported only V_ave_ during systolic time at the left ventricular septum, we hypothesize that the development of more detailed myocardial damaging process becomes clear by analyzing the continuous temporal relationship with V_ave_, V_max_, and color image. The nature of cardiac tissue that exhibits low levels of antioxidative enzymes, such as superoxide dismutase and catalase, make it more susceptible to redox oxidative stress (ROS) generation and accumulation of oxidative stress. The major mechanism of chemotherapy‐induced cardiotoxicity involves the generation of ROS. In turn, elevated ROS causes cellular damage and alternation responses 38. Therefore, measurement with noninvasive the PTM, which is measured with small‐amplitude velocity signals of less than a few micrometers, before and after chemotherapy helps to prevent cardiotoxicity.

Careful observation is required to detect decreases of more than 1.0 (m/s)/m in such patients to consider treatment delay or cancelation in order to avoid lethal cardiotoxicity. To circumvent the development of HF, early detection of drug‐induced changes in cardiac function is essential for preventive intervention. Careful surveillance using the PTM could ensure early detection and timely management of such cardiotoxicity.

## Conclusion

The PTM was superior to echocardiography for early, noninvasive detection and intermediate‐term monitoring of left ventricle systolic function associated with anthracycline chemotherapy in patients with hematologic malignancies. We found that the PTM is a valid laboratory procedure that may help avoid progression to serious cardiomyopathy.

## Conflict of Interest

The authors declare no conflicts of interest.
